# Social-Economic Environments and Depressive Symptoms in Community-Dwelling Adults: A Multi-Level Analysis for Two Nationwide Datasets in Taiwan

**DOI:** 10.3390/ijerph18147487

**Published:** 2021-07-13

**Authors:** Susan C. Hu, Yu-Hsuan Tsai, Der-Chiang Li, Wan-Chen Hsu, Nuan-Ching Huang

**Affiliations:** 1 Department of Public Health, College of Medicine, National Cheng Kung University, Tainan City 701, Taiwan; shuhu@mail.ncku.edu.tw (S.C.H.); jenny13929@gmail.com (W.-C.H.); 2 Department of Computer Science and Information Engineering, National Cheng Kung University, Tainan City 701, Taiwan; awedxzs3@gmail.com; 3 Department of Industrial and Information Management, National Cheng Kung University, Tainan City 701, Taiwan; lidc@mail.ncku.edu.tw; 4 Healthy City Research Center, Innovation Headquarter, National Cheng Kung University, Tainan City 701, Taiwan

**Keywords:** social-economic features, social environments, economic environments, adults, depression, multi-level analysis

## Abstract

Most studies have focused on factors associated with depression at the individual level, and evidence on ecological models linking social-economic features with depression is rare in Taiwan. This study aimed to use multi-level analysis to explore the effects of social-economic environments on depressive symptoms among Taiwanese adults. The 2009 National Health Interview Survey (NHIS) and the Age-Friendly Environments database were linked in this study. A total of 6602 adults aged 20 years and older were included in the analysis. A Chinese version of the 10-item CESD was used as the outcome measure. Three social indicators (population density, divorce rate, and crime rate) and three economic indicators (unemployment rate, per capita disposable income, and per capita government expenditures) at the ecological level were examined. Results showed that two social environments and two economic features were significantly associated with depressive symptoms. However, the effects of these factors were different by gender and age groups. The economic environments were critical for males and young adults aged 20–44 years old, whereas the social environments were significant for females and middle-aged and older adults. Intervention efforts for depression prevention should integrate ecological approaches into the effects of social-economic environments on depressive symptoms.

## 1. Introduction

Depression is a common mental health disorder worldwide. It significantly affects physical health and daily life including declining physical function and quality of life, falling tired or distracted, and having sleeping or eating problems. To estimate, until 2017, around 4.4% of the population worldwide (with more than 300 million) were affected by depression [1].

Previous studies have shown that females, young adults, low level of education, without a spouse, low income, health illness, and lack of social and physical activity are the potential risk factors of being depressed [2,3]. Other research has provided evidence of the relationships between social-ecological environments and health outcomes [4,5]. For example, population density, economic deprivation, neighborhood violence, crime, and safety are highly correlated to depression [6,7]. Thus, multi-level analyses are often suggested to explore the effects of environmental features on depression symptoms [8,9].

However, most of the previous studies exploring factors associated with depression in Taiwan have focused more on individual characteristics and indicated that females, unemployment, low education level, and poor health were the risk factors of depression [10,11,12]. Scarce studies have examined the relationships between social-economic environments and depression. Only two recent studies by Liu et al. (2020) and Liu (2021) were found to investigate the effects of social-economic environments on health, where their outcome variables were dementia and mortality, while the social-economic indicators differed from a case to another [13,14].

In fact, the social-economic environments vary in different countries, and the impacts on mental health are inconsistent [15,16,17]. Behanova et al. (2013) examined the association between the neighborhood unemployment rates and mental health problems by a direct comparison of a Central and a Western European country, Slovakia and the Netherlands, respectively. Both countries showed a similar prevalence of mental health problems (39.3% vs. 39.2%, respectively). However, results showed that a gradient relationship of area unemployment and mental health problem was observed in the Netherlands, instead of a smooth pattern in Slovakia. In addition, the difference between Slovakia and the Netherlands cannot be interpreted by individual-level socioeconomic characteristics. A possible explanation for the result may be that both countries are at different stages of economic development. Slovakia has been transformed from a formerly centrally planned economy into a market economy and joined the European Union in 2004. The Netherlands is one of the founding members of the European Union and its GDP was the top 17th in the world in 2019. Furthermore, the unemployment rate in the Netherlands (5.3%) was lower than in Slovakia (14%) [15]. Hence, economic growth situation and operate strategies in different countries impact differently on mental health.

Taiwan is a small economy that used to be known as one of the four Asian tigers with different social and economic environments from Western countries. Minimal studies have explored the associations between social-economic environments and depression in Taiwan. The purpose of the study was to use multi-level analysis to examine the effects of the social-economic features on depressive symptoms among Taiwanese adults.

## 2. Materials and Methods

Two nationwide datasets including the 2009 National Health Interview Survey (NHIS) and the Age-Friendly Environment database-city/county level were used with a multi-level design in this cross-sectional study. The 2009 NHIS is a representative survey of each county and municipality in Taiwan using multi-stage stratified systematic sampling methods with a probability proportional to population size. A total of 25,636 participants (response rate of 83.96%) were interviewed, covering questions including demographic characteristics, health status, health services utilization, health literacy, and lifestyles, and was divided into three age groups: under 12 years (3531 samples), 12–64 years (19,201 samples), and over 65 years of age (2904 samples). 

**Figure 1:**
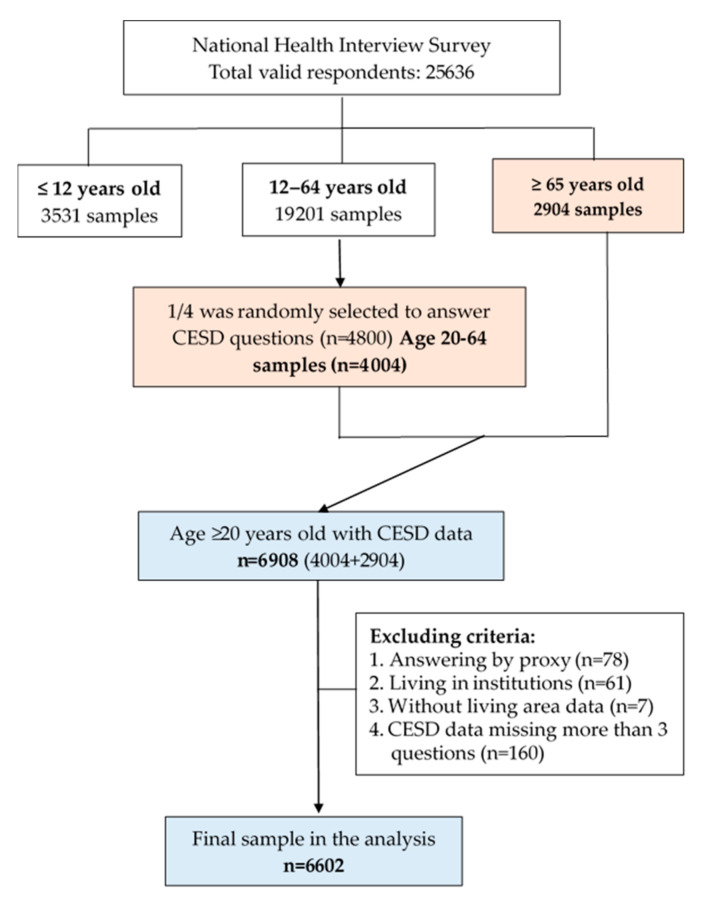
The procedure of participant enrollment.

Figure 1 presents the procedure of participant enrollment. Since four additional module questionnaires were added in the age group of 12–64, only one-quarter of the participants were randomly selected to answer one module questionnaire. Hence, 4404 adults aged 20–64 years old answered the Chinese version of the Center for Epidemiological Studies Depression Scale (CESD). Thus, a total of 6908 adults aged 20 years and older were initially recruited in the study, and 6602 adults ≥ aged 20 years were finally included in the analysis after excluding those living in institutions (*n* = 61), not self-reported (*n* = 78), without living area data (*n* = 7), and missing more than three questions of CESD (*n* = 160).

### 2.1. Measurement

#### 2.1.1. Outcome Variable: Depressive Symptoms

This study used a 10-item Chinese version of the CESD abbreviated from the original English version to measure depressive symptoms [18,19]. This scale has been tested with good validity and reliability in the Taiwan Longitudinal Study on Aging [20] and implemented in other studies [21,22]. The severity of each item was scored from 0 (rarely or none of the time) to 3 (most or all of the time). The total score ranges from 0 to 30, and a recommend cutoff of ≥10 is suggested to detect depressive symptoms [20,23]. Accordingly, both the continuous (CESD score) and dichotomous (depression yes/no) outcomes of the CESD scale were adopted in this study.

#### 2.1.2. Study Variables: Social and Economic Environments

Variables of the social-economic environments were obtained from the Age-Friendly Environment Dataset at the city-county level in Taiwan [24]. All indicators in this dataset were collected from the open-access data and national surveys from governments. 

First of all, eight environmental factors at the ecological level in the same year of NHIS were examined in the analysis. Namely, social factors included population density (persons/km^2^), divorce rate (%), crime rate (events per 100,000 persons), and educational level (% of college and above). Economic factors consisted of the unemployment rate (%), per capita disposable income (NT$), per capita government expenditures (NT$), and the percentage of low-income households (%).

Then, the Spearman correlation and the variance inflation factor (VIF) were assessed to prevent multicollinearity before performing multi-level regression models. Here, we found that educational level showed a high correlation (coefficient > 0.7) with other variables including population density, crime rate, and per capita disposable income. In addition, the VIF of the low-income households (%) was greater than 4 in the models. Thus, we deleted these two ecological variables in the analysis.

Finally, only six social-economic factors were examined in this study including three social environments (population density, divorce rate, and crime rate) and three economic factors (unemployment rate, per capita disposable income, and per capita government expenditures). As the skewness still appeared after using several transformation methods, we thus categorized the study variables into groups to lower the misclassification for normality. Thus, all study variables were divided by tertiles (low, medium, and high) and the lowest served as the reference group [13].

#### 2.1.3. Control Variables

Control variables at the individual level in this study included gender, age (20–44, 45–64, and ≥65 years), spouse (yes/no), level of education (≤6 years, 7–9 years, and ≥10 years), employment status (yes/no), religion (yes/no), smoking (yes/no), alcohol consumption (yes/no), physical activity (yes/no), and self-rated health (good/fair/bad). Furthermore, we used the question “have you engaged in physical activity in the past two weeks?” to define whether adults engage in physical activity and a question, “have you drunk alcohol in the past seven days?” to define alcohol consumption. Self-rated health was measured by the question “In general, how would you rate your health today?” with choices from “(5) very good” to “(1) very bad”, and was then grouped into three levels as good, fair, and bad in the analysis. 

### 2.2. Statistical Analysis

All variables were merged together for the individual and ecological data. The analysis was performed using SAS 9.4 software (SAS Institute, Cary, NC, USA). Statistical methods included descriptive analysis, bivariate analysis (Chi-square test and t-test), and multi-level analysis. 

Two different models were built to examine the relationships between social-economic environments and depression. First, for the continuous outcome (CESD score), two levels of random intercept **linear regression** models were conducted to detect the effect of individual factors and social-economic features on the CESD score with the regression coefficient (β). A *p*-value of <0.05 was recognized as statistically significant. The SAS procedure MIXED was performed for multi-level regression models (individual at level 1 nested within townships at level 2) [25]. 

Second, for the dichotomous outcome (CESD ≥ 10), two levels of random intercept **logistic regression** models were conducted to examine the effect of individual and environmental factors on the risk of being depressed with the odds ratios (OR) and 95% confidence intervals (CI) by the SAS procedure GLIMMIX for the categorical outcome procedure [26]. In addition, stratified analyses were also performed to detect the difference in gender and age groups.

## 3. Results

### 3.1. Characteristics of Participants

Table 1 shows the characteristics of participants in the study. The mean age of respondents was 53.94 ± 19.29, the mean score of CESD was 5.11 ± 4.32, and 15.1% showed depressive symptoms (CESD ≥ 10). Except for religion and alcohol consumption, all other variables were significantly related to a higher CESD score including being female, younger group (20–44 years old), less than six years of education, without a spouse, unemployed, a smoker, not engaging in physical activity, and with bad self-rated health. These variables were also found to be significantly related to depressive symptoms defined as CESD score ≥10.

### 3.2. Social-Economic Environments in Cities and Counties (n = 23)

Table 2 lists the descriptive statistics of the social-economic environments in the 23 cities and counties in Taiwan. The mean, standard deviation, min, max, and median of the six indicators are shown in the table. For example, the population density differed significantly from min 66.1 to the max 9947.8. The divorce rates were from 4.5% to 9.4%, and the mean unemployment rate was 5.8%, ranging from 5.7%~6.0%. 

### 3.3. Multi-Level Analysis by Gender

Table 3 summarizes the results of the multi-level analysis stratified by gender. In the continuous outcome model (CESD score), only the unemployment rate illustrates a dose-response relationship in males. In contrast, population density in the medium group was significantly associated with a higher CESD score for females. In the cutoff model (CESD ≥ 10), the results showed that male adults living in cities/counties with an unemployment rate in both medium and high groups (OR = 1.78, 2.34; 95% CI = 1.13–2.81, 1.42–3.86, respectively) and per capita government expenditures in the high group (OR = 1.75; 95% CI = 1.21–2.53) had higher chance of being depressed compared with those in the low group. Nevertheless, female adults living in cities/counties with a population density in the medium group (OR = 1.54, 95% CI = 1.08–2.21) and divorce rate in both the medium and high groups had a higher risk of being depressed (OR = 1.95, 1.53; 95% CI = 1.14–3.36, 1.00–2.34, respectively) compared with those in the low group. 

### 3.4. Multi-Level Analysis by Age Groups

Table 4 exhibits the ecological factors associated with the depressive symptoms by age group after controlling all individual factors. In the continuous outcome model (CESD score), the results showed that for young adults aged 20–44 years, the unemployment rate and per capita government expenditures were positively related to higher CESD scores. However, none of the six social-economic environments at the ecological level were significant for middle-aged (45–64 years) and older adults (≥65 years). 

In the cutoff model (CESD ≥ 10), similarly, young adults aged 20–44 years living in areas with the unemployment rate in the medium and high groups (OR = 1.70, 1.73; 95% CI = 1.11–2.62, 1.05–2.84, respectively) and per capita government expenditures in the high group (OR = 1.57; 95%CI = 1.10–2.26) had a higher chance to be depressed, compared with those in the low group. However, the results were significantly different in the other two age groups. For example, adults aged 45–64 years living in cities/counties with the divorce rate in the medium group had 3.27 times (OR = 3.27, 95% CI = 1.03–10.42) as many chances of being depressed compared with those in the low group. Nevertheless, opportunities of being depressive for older adults aged 65 years and above were related to population density in the medium group (OR = 1.87, 95% CI = 1.18–2.96) and divorce rate in both medium and high groups (OR = 2.58, 1.77, 95% CI = 1.27–5.24, 1.02–3.06, respectively).

## 4. Discussion

This study found that two social factors (population density, divorce rate) and two economic features (unemployment rate, per capita government expenditures) were significantly associated with depressive symptoms among Taiwanese adults. However, the effects of these factors were different by gender and age groups. For gender difference, the population density and divorce rates were significantly associated with females, while the unemployment rate and government expenditures were positively related to males. For age-group differences, economic factors such as the unemployment rate were crucial for the young generation (aged 20–44 years). In contrast, social factors such as divorce rate and population density were more notable for middle-aged (aged 45–64 years) and older generations (aged 65 years and over).

### 4.1. Social Environments

This study examined three social factors including population density, divorce rate, and crime rate in the analysis. Only the crime rate was not associated with depressive symptoms in the study, which is not consistent with past studies. For example, a study from the United Kingdom (UK) indicated that the crime rate was related to mental distress for residents aged 50 and above, and the effect was more substantial for females [27]. They also indicated that property crime primarily caused considerable mental distress for residents. However, the crime rate in Taiwan is much lower than that in other Western countries, so the crime rate may not be a risk factor of being depressed among adults in Taiwan. 

In this study, the population density in the medium group played a critical role in increasing CESD score and being depressed, especially for female and older adults aged 65 years and over. Associations of depressive symptoms and urban–rural living environments have been examined in several previous studies, although the result is not consistent. The influences of population density on mental health are complicated and nonlinear because they would interact with other physical and social-economic environments [28,29]. For instance, a study from the UK indicated that people older than 75 years living in the highest population density and intermediate-lower density areas were related to the high opportunity of depression compared with the lowest density areas [29].

The divorce rate was also found here to be a risk factor of being depressed, especially for females and adults aged 45 years and over. A longitudinal study using community-based data from the New Haven Epidemiologic Catchment Area program on respondents aged 18–60 years found that the prevalence of major depression was 3.7 times higher among married females than among their male counterparts [30]. However, another study found that the depression effect in males was more sensitive to divorce or separation and work problems, but females were more sensitive to getting along with people [31]. 

### 4.2. Economic Environments

We examined three economic factors at the ecological level in this study: unemployment rate, per capita disposable income, and per capita government expenditures. Only disposable income was not associated with depressive symptoms, regardless of continuous or dichotomous outcomes. However, the unemployment rate and per capita government expenditures were the critical factors of depressive symptoms, especially for males and young adults (20–44 years). Such findings were not consistent with previous studies [15,16,17].

For example, a study from Behanova et al. (2013) comparing urban-area unemployment on mental health in Slovak and Dutch cities showed different results [15]. For instance, a gradient relationship between the area unemployment rate and mental health problems was observed in the Netherlands but absent in Slovakia. The finding in the Netherlands is similar to our study, showing that the effect of unemployment was more significant for males. Traditionally, males and young adults (aged 20–44 years) are the primary support for a family’s financial security, thus being more sensitive to the pressure of the employment situation. 

Another nationwide cross-sectional study in Korea conducted by Lee and Park (2015) found that living in a higher mean income of communities would increase the risk of being depressed compared with people in the lowest level, even by gender and age [8]. However, no association was found between city-level median income and mental disorders in China [32], which is similar to our results. Other studies that used indicators of income inequality such as the GINI index or GINI coefficients also expressed inconclusive results [33,34]. These inconsistent results may vary with variable definitions or measurements at different levels and other contexture factors such as economic growth. Hence, research about the above factors is warranted in the future.

One unanticipated finding was that government expenditure in the high group significantly increased the risk of being depressed than in the low group, especially in males and young adults (aged 20–44 years). Little research has examined the association between government expenditures and mental health. A recent study from Reeves et al. (2016) in the United Kingdom exploring the relationship between government housing benefit and depression symptoms in low-income households revealed that reducing housing support to low-income people in rental houses would increase the prevalence of depressive symptoms [35]. Similarly, Matsubayashi et al. (2020) found that decreasing local government spending would worsen the suicide rate in Japan, especially for males, when the unemployment rate increased [36]. However, “government expenditure” is an aggregated indicator covering expenditures in various categories and items such as infrastructure building, repairing, environmental protection, social welfare, and related activities. Hence, it would face a challenge with the aging population. 

### 4.3. Gender and Age Difference

Gender and age were found significantly different in this study. The divorce rate was crucial to depressive symptoms for females and older adults. A possible explanation for the result may be from the pressure of discrimination and insecurity [37,38]. To a certain extent, economic, and life stability is secure from marital relationships for females and older adults. In addition, both categories are usually vulnerable in social-economic status and the labor market. Thus, the stress of living in disadvantaged areas, along with their actual difficult life situation (such as divorce, with children, work–life unbalance) and fewer economic choices or low salary, would create an opportunity for mental illness such as depression [37,38].

In contrast, the economic environment is a significant factor for depressive symptoms of males and young adults. These results also appear in previous studies [8,39]. A possible explanation might be that males traditionally play a fundamental role in household finances. Hence, regional economic phenomenon and prosperity would affect their employment pressure, investment, and financial situation. In addition, young adults also faced the pressure of the economic boom and the choices of career or industry involvement. Accordingly, they are susceptible to local economic factors.

### 4.4. Contributions and Limitations

In Asia, little research has explored the effects of social-economic environments on depressive symptoms. Using Taiwan as an example, we linked a nationally representative survey and social-economic data at the city-county level, providing better evidence than previous studies. We found that the association between social-economic environments and depression was different by gender and age.

However, there were some limitations in the study. First, this was a cross-sectional study that only examined the association between social-economic environments and depressive symptoms, but could not explain the causation of related factors. Further longitudinal research is needed to confirm the causality in the future. Second, social-economic factors in this study were mainly obtained from the open governmental data. Some indicators do not provide detailed information, so the relationships need to be clarified in future studies.

## 5. Conclusions

This study provides preliminary evidence that the social-ecological features are associated with depressive symptoms in community-dwelling adults. The effects of social-economic environments on depressive symptoms varied across different gender and age groups. The economic environments were critical for male and young adults aged 20–44 years old, whereas the social environments were significant for females and middle-aged and older adults. Hence, intervention efforts to improve depressive symptoms should integrate a social-ecological approach into the effects in different gender and age groups.

## Figures and Tables

**Table 1:** Characteristics of participants in the study (*n* = 6602).

	Total		CESD Score			CESD ≥ 10		
	*n*	%	Mean	SD	*p*-Value	*n*	%	*p*-Value
Total	6602	100.0	5.11	4.32		1000	15.2	
Gender					**<0.0001**			**<0.0001**
Male	3000	45.4	4.77	4.10		388	12.9	
Female	3602	54.6	5.38	4.47		612	17.0	
Age (year)					**<0.0001**			**0.0005**
20–44	2426	36.8	5.52	4.12		398	16.4	
45–64	1569	23.8	4.56	4.10		190	12.1	
65+	2607	39.5	5.05	4.59		412	15.8	
Level of education					**0.0365**			**0.0005**
≤6 years	2562	38.9	5.27	4.69		440	17.2	
7–9 years	827	12.6	4.97	4.21		122	14.8	
≥10 years	3196	48.5	4.99	4.01		431	13.5	
Spouse					**<0.0001**			**<0.0001**
Yes	4054	61.5	4.63	4.16		509	12.6	
No	2543	38.5	5.86	4.46		490	19.3	
Employment					**<0.0001**			**<0.0001**
Yes	3291	49.9	4.89	3.95		432	13.1	
No	3309	50.1	5.32	4.65		568	17.2	
Religion					0.1381			0.1623
Yes	4849	73.5	5.06	4.35		716	14.8	
No	1751	26.5	5.24	4.22		283	16.2	
Smoking					**0.0002**			**0.0033**
Yes	1936	29.3	5.41	4.45		332	17.1	
No	4665	70.7	4.98	4.26		667	14.3	
Alcohol consumption					0.1385			0.3881
Yes	1188	18.0	4.93	4.33		170	14.3	
No	5405	82.0	5.14	4.31		827	15.3	
Physical activity					**<0.0001**			**<0.0001**
Yes	3383	51.6	4.34	3.76		344	10.2	
No	3180	48.5	5.87	4.66		639	20.1	
Self-rated health					**<0.0001**			**<0.0001**
Good	3314	50.3	3.89	3.44		233	7.0	
Moderate	2532	38.5	5.58	4.16		449	17.7	
Bad	740	11.2	8.96	5.64		318	43.0	

The bold number means statistically significant (*p* < 0.05).

**Table 2:** Descriptive statistics of the social-economic environment in the level of cities and counties (*n* = 23).

Variables (Unit)	Mean	SD	Median	Min	Max
Population density (persons/km^2^)	2239.1	2963.6	758.4	66.1	9947.8
Divorce rate (%)	6.9	1.2	6.7	4.5	9.4
Crime rate (events/100,000 persons)	1503.8	395.8	1444.2	733.9	2187.4
Unemployment rate (%)	5.8	0.1	5.8	5.7	6.0
Per capita disposable income (NT$) *	249,467	41,931	233,616	207,073	387,053
Per capita government expenditures (NT$) *	41,511	12,846	42,236	24,296	79,453

* 1US$ ≈ 30NT$.

**Table 3:** Multi-level analysis for factors associated with the depressive symptoms of adults by gender.

	CESD Score *				CESD ≥ 10 *			
	Male *		Female *		Male *		Female *	
	β	*p*	β	*p*	OR	95% CI	OR	95% CI
Intercept	4.10	0.0001	4.22	0.0001				
Population density (ref: low) ^#^								
Medium	0.74	0.0572	**0.83**	**0.0333**	1.41	0.90–2.21	**1.54**	**1.08–2.21**
High	−0.04	0.9249	−0.31	0.4233	1.13	0.66–1.93	0.82	0.53–1.25
Divorce rate (ref: low) ^§^								
Medium	0.68	0.1903	0.57	0.2446	1.33	0.66–2.67	**1.95**	**1.14–3.36**
High	0.60	0.1584	0.39	0.3280	1.15	0.68–1.95	**1.53**	**1.00–2.34**
Criminal rate (ref: low) ^ҍ^								
Medium	0.15	0.6938	0.70	0.0824	0.87	0.52–1.45	1.34	0.90–1.99
High	−0.12	0.7944	0.34	0.4717	0.83	0.44–1.57	1.03	0.63–1.69
Unemployment rate (ref: low) ^§^								
Medium	**0.80**	**0.0230**	0.64	0.0659	**1.78**	**1.13–2.81**	1.17	0.84–1.63
High	**0.92**	**0.0270**	0.39	0.3121	**2.34**	**1.42–3.86**	1.12	0.75–1.66
Disposable income (ref: low) ^¶^								
Medium	−0.19	0.5778	−0.23	0.5051	0.74	0.47–1.18	0.80	0.55–1.17
High	0.33	0.4137	0.23	0.5530	1.08	0.64–1.80	0.88	0.57–1.34
Government expenditures (ref: low) ^¶^								
Medium	0.26	0.4886	0.67	0.0936	0.93	0.59–1.46	1.28	0.88–1.86
High	0.56	0.0769	0.28	0.3650	**1.75**	**1.21–2.53**	1.17	0.85–1.59
Fit Statistics								
−2 Res Log Likelihood	16,120.7		19,816.8		1929.1		2711.2	
AIC	16,124.7		19,820.8		1979.1		2761.2	
BIC	16,127.0		19,823.1		2007.4		2789.6	

OR: odds ratio, CI: confidence interval. The bold number means statistically significant (*p* < 0.05). Unit: ^#^ (persons/ km^2^), ^§^ (%), ^ҍ^ (events /100,000 persons), ^¶^ (Per capita dollars, NT$). * Adjusted by individual factors including age, level of education, spouse, employment, religion, smoking, alcohol consumption, physical activity, and self-rated health.

**Table 4:** Multi-level analysis for factors associated with the depressive symptoms of adults by age groups.

	CESD Score *						CESD ≥ 10 *					
	20–44 years		45–64 years		≥65 years		20–44 years		45–64 years		≥65 years	
	β	*p*	β	*p*	β	*p*	OR	95%CI	OR	95%CI	OR	95%CI
Intercept	2.01	0.0312	3.92	0.0002	3.07	0.0061						
Population density (ref: low) ^#^												
Medium	0.50	0.1152	0.78	0.0594	1.09	0.0725	1.34	0.88–2.04	1.79	0.87–3.70	**1.87**	**1.18–2.96**
High	0.04	0.8972	−0.29	0.4907	−0.40	0.5266	1.01	0.62–1.64	1.15	0.50–2.66	0.67	0.38–1.18
Divorce rate (ref: low) ^§^												
Medium	0.40	0.3494	0.85	0.1336	1.00	0.2120	1.41	0.75–2.65	**3.27**	**1.03–10.42**	**2.58**	**1.27–5.24**
High	0.44	0.2130	0.52	0.2401	0.76	0.2437	1.32	0.81–2.14	2.25	0.93–5.46	**1.77**	**1.02–3.06**
Criminal cases (ref: low) ^ҍ^												
Medium	0.46	0.1797	0.26	0.5146	0.47	0.4332	1.07	0.66–1.72	0.80	0.37–1.72	1.19	0.72–1.97
High	0.23	0.5769	−0.03	0.9446	0.09	0.9038	0.99	0.55–1.79	0.63	0.24–1.66	0.97	0.51–1.84
Unemployment rate (ref: low) ^§^												
Medium	1.15	**0.0021**	0.56	0.1060	0.46	0.3626	**1.70**	**1.11–2.62**	1.08	0.60–1.93	1.24	0.82–1.87
High	0.99	**0.0129**	0.44	0.2737	0.41	0.4880	**1.73**	**1.05–2.84**	1.46	0.71–3.00	1.30	0.81–2.09
Disposable income (ref: low) ^¶^												
Medium	−0.30	0.3374	−0.01	0.9726	−0.35	0.5207	0.77	0.49–1.21	0.69	0.33–1.45	0.67	0.42–1.09
High	0.00	0.9943	0.49	0.2624	0.30	0.6354	0.86	0.53–1.38	0.86	0.37–2.02	1.09	0.62–1.91
Government expenditures (ref: low) ^¶^												
Medium	0.91	**0.0168**	0.10	0.7874	0.28	0.6357	1.49	0.95–2.33	0.89	0.46–1.71	0.98	0.62–1.56
High	0.63	**0.0294**	−0.08	0.8009	0.44	0.3702	**1.57**	**1.10–2.26**	1.05	0.62–1.78	1.35	0.92–2.00
Fit Statistics												
−2 Res Log Likelihood	13,310.7		8415.8		14,151.1		1940.8		934.2		1737.2	
AIC	13,312.7		8417.8		14,155.1		1988.8		982.2		1785.2	
BIC	13,313.8		8419.0		14,157.3		2016.1		1009.4		1812.4	

OR: odds ratio, CI: confidence interval. The bold number means statistically significant (*p* < 0.05). Unit: ^#^ (persons/ km^2^), ^§^ (%), ^ҍ^ (cases /100,000 persons), ^¶^ (Per capita dollars, NTD). * Adjusted by individual factors including gender, level of education, spouse, employment, religion, smoking, alcohol consumption, physical activity, and self-rated health.

## Data Availability

The individual-level data presented in this study can be obtained from the Data Science Center, Ministry of Health and Welfare, Taiwan with the permission from the Health Promotion Administration, Ministry of Health and Welfare in Taiwan. The ecological-level data presented in this study are available on request from the corresponding author.
